# Removal of the Metal Ion Lead II from Aqueous Effluents Using the Stem Bark of *Ximenia americana* L

**DOI:** 10.1021/acsomega.5c01460

**Published:** 2025-09-05

**Authors:** Roseni da Silva Cardoso, Carliane de Oliveira de Souza, Daniel Bernardes Silva, Lucia Raquel de Lima, Jorge Marcell Coelho Menezes, Mira Raya Paula de Lima, Hiago de Oliveira Gomes, Yannice Tatiane da Costa Santos, Francisco José de Paula Filho, Henrique Douglas Melo Coutinho, Raimundo Nonato Pereira Teixeira

**Affiliations:** † Department of Biological Chemistry, 226206 Regional University of Cariri, R. Cel. Antonio Luis 1161, Crato 63105000, CE, Brazil; ‡ Science and Technology Center, Federal University of Cariri, Av Ten. Raimundo Rocha 1639, Juazeiro do Norte 63048-080, Ceará, Brazil; § Federal Institute of Education, Science and Technology of Ceará, Campus Juazeiro do Norte, Av. Plácido Aderaldo Castelo 1646, Juazeiro do Norte 63047040, Ceará, Brazil; ∥ Federal Institute of Education, Science and Technology of Ceará, Campus Iguatu, Rodovia Iguatu/Várzea Alegre S/N, Iguatu 63500000, Ceará, Brazil

## Abstract

In recent years, the pollution and contamination of water with heavy metals have posed significant risks to human health and have compromised aquatic life. As a result, the use of biosorbent materials to remove these metals from effluents has emerged as an alternative method for their remediation. This study evaluated the adsorption potential of *Ximenia americana* L. stem bark (in natura) for the removal of Pb^2+^ from aqueous solutions. The process involved the collection, washing, drying, and grinding of the shell, resulting in a powder used for characterization techniques, kinetic assays (conducted at intervals from 1 to 120 min), isotherms using metal solutions ranging from 40 to 600 mg L^–1^, and adsorption thermodynamics at temperatures of 278.15, 298.15, and 318.15 K. Desorption assays showed that the adsorbent is reusable, maintaining its efficiency in the adsorption process without significant losses. In coexisting ion tests, it was observed that the evaluated ions did not compete for the same active sites, indicating selectivity in adsorption. The point of zero charge (PZC) of the material was determined to be 6.0, suggesting that its surface becomes positively charged at pH values below this point, favoring the adsorption of negatively charged species. In dosage tests, the amount of material used did not directly influence the adsorption process. The material was characterized using scanning electron microscopy (SEM), which revealed significant morphological differences among the bark samples, including variations in particle shape and size; these differences may affect adsorption capacity. After treatment with Pb^2+^ ions, crystals were observed on the surface of the bark, suggesting the formation of complexes between the metal ions and biomass components, potentially increasing the material’s affinity for the metal. Fourier-transform infrared spectroscopy (FT-IR) showed changes in absorbance bands, highlighting the presence of compounds such as aromatics, carbonyls, and esters. X-ray fluorescence (XRF) analysis revealed a significant difference in Pb^2+^ ion retention between treated (13.12%) and untreated bark (0.0%). The fact that the raw shells contained no residual metals indicates a promising potential for metal ion removal processes without the need for additional chemical treatments. The adsorption kinetics followed a pseudo-second-order kinetic model, with an adjusted *R* ^2^ of 0.999. Equilibrium adsorption data were analyzed and best fitted the Sips adsorption model, with a *q*_max_ of 138.20 mg g^–1^, and the best fit achieved at 278.15 K with an *R* ^2^ value of 0.998. This indicates that the adsorption process is based on chemical interactions, demonstrating high efficiency in capturing Pb^2+^ and highlighting the strong affinity of the material for the metal ion, as well as its potential for environmental decontamination. Thermodynamic analysis showed that the reaction was spontaneous at all studied temperatures, with Δ*G*° values of −3.21, −9.01, and −14.8 kJ mol^–1^. The process was endothermic, with Δ*H*° = 77.4 kJ mol^–1^, and showed increased system disorder, as indicated by Δ*S*° = 0.29 kJ mol^–1^ K^–1^. In view of these findings, this study contributes to the development of sustainable alternatives for environmental decontamination and highlights *X. americana* as a promising natural resource for the treatment of wastewater and industrial effluents, its bark, although little explored, presents high efficiency, low cost, selectivity and reuse, characteristics that position this biosorbent as a viable alternative, compared to conventional technologies such as activated carbon, membranes and nanomaterials.

## Graphical abstract

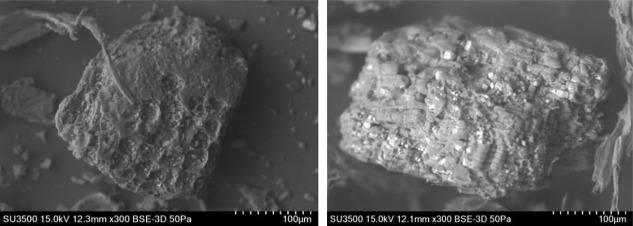

## 1 Introduction

In recent decades, inappropriate human activities have threatened the quality of water resources, causing damage to both the environment and human health.^1^ Increased industrialization, lack of proper treatment, and the inappropriate disposal of solid and liquid waste from industrial processes, agriculture, and domestic sewage are the main sources of environmental contamination.^2^

According to Rios et al.,^3^ aquatic pollution refers to any biological, physical, or chemical modification that disrupts or transforms an ecosystem, altering its animal and plant composition. Among the various pollutants, heavy metals stand out due to their presence in multiple forms in nature and their high toxicity, which allows them to easily accumulate in both aquatic and terrestrial environments.^4^

Heavy metals is a collective term applied to a group of metals or metalloids with an atomic density greater than 6 g cm^–3^.^5^ These substances are nondegradable and tend to accumulate in the environment, where they manifest their toxicity.^6^ Metals become toxic to humans when their concentrations exceed permitted levels. Lead is one of the most common heavy metals found in wastewater and is known to be toxic to both humans and plants.^7^ Industrial processes that contribute to anthropogenic lead pollution include metallurgy, battery manufacturing, printing operations, lead mining and smelting as well as ceramics and glass production, as stated by Liu et al.^8^

Based on the toxicity of heavy metals, the U.S. Environmental Protection Agency (EPA) and the World Health Organization (WHO) have established a maximum permissible limit for lead ions in drinking water of 0.05 mg L^–1^.^9^ Lead is highly toxic, accumulates easily, and is readily retained in human organs. The most sensitive targets include the developing nervous system, as well as the hematological, cardiovascular, and renal systems. It can also impair cognitive function in both children and adults, with children being particularly vulnerable.^10^

Kumar^11^ reported that numerous studies have shown that lead exposure negatively affects human reproductive health and adversely affects pregnancy outcomes. These findings have been known for some time, and various stakeholders worldwide are making efforts to reduce, restrict, or eliminate human exposure to lead. Reports indicate that lead can induce infertility and hormonal imbalances in both sexes, reduce libido, affect spermatogenesis, disrupt the ovarian cycle in women, and cause various fertility-related and pregnancy complications.

Therefore, effluents containing heavy metals should not be discharged into public sewer systems for treatment alongside domestic sewage.^4^ Conventional methods used to remove heavy metals from industrial effluents include chemical reduction, precipitation, filtration, ion exchange, and adsorption. These techniques aim to reduce the concentration of metal ions in wastewater to levels compliant with regulatory standards.^12,13^

Adsorption is a highly effective and low-cost alternative that involves the accumulation of a substance at the interface between two phases typically a solid surface and a surrounding solution. Natural adsorbents such as lignocellulosic materials can be employed in this process. These biosorbents are notable for being abundant, renewable, and economically viable, in addition to possessing good adsorption capacity.^13,14^ Although recent advances have promoted the use of nanomaterials, membranes, and activated carbon, these technologies still face limitations such as insufficient selectivity, challenges in material regeneration, and high operational costs.^14–16^

As shown in Table 1, natural biosorbents such as the bark of *Ximenia americana* L. offer significant advantages over conventional methods, including high selectivity, reusability, and reduced cost. This comparison reinforces the proposition of using alternative, sustainable materials as an effective solution for the treatment of effluents contaminated by heavy metals.

*X. americana* L. belongs to the Olacaceae family and is commonly known as wild plum, among other regional names. According to Fernandez and Bezerra,^17^ the wild plum is part of the shrub vegetation of the Caatinga and is one of its dominant species. During the dry season, when most species in the Caatinga lose their leaves, this plant remains fully green, making it a drought-resistant species. Its fruiting period is very short, typically occurring between December and January.

In a review study, Monte^18^ compiled chemical and pharmacological information on wild plum, updating relevant data from the past decade. Its phytochemical composition includes various functional groups such as phenols, flavonoids, carbonyls, hydroxyls, carboxyls, and esters, which contribute to its biological activities and adsorption capacity. These chemical groups promote interaction and complexation with metal ions, increasing the efficiency of the biomass in removing contaminants. Documented biological activities include antimicrobial and antifungal (leaves, root, bark, and stem), analgesic (stem bark and leaves), antipyretic (stem bark), antitrypanosomal (stem bark), antiviral (stem bark), antioxidant, as well as hematological and hepatoprotective effects (leaves, stem bark, and root).

This study aims to explore the potential of *X. americana* L. bark in removing Pb^2+^ ions from wastewater and industrial effluents. Considering its natural abundance in the state of Ceará, this research highlights the potential use of this biomass, especially due to its phytochemical profile, which can significantly contribute to the adsorption process. This approach reinforces the relevance of *X. americana* as a sustainable and promising alternative for the treatment of contaminated water.

## 2 Methods

### 2.1 Preparration of Biosorbent

The bark of *X. americana* L. (wild plum) was collected in the municipality of Caririaçu, Ceará, Brazil. The material was washed with distilled water to remove impurities, then dried in an oven at 313.15 K for 48 h to eliminate moisture. After drying, the bark was crushed and sieved to obtain particles with a diameter of 0.0018 cm (180 μm).

### 2.2 Point of Zero Charge

We adopted the methodology described by Regalbuto and Robles,^19^ known as the “11-point experiment,” which involved the preparation of H_2_SO_4_ and NaOH solutions, both at 0.1 mol L^–1^, to obtain a pH range from 1 to 13, with adjustments made as necessary. Then, 10 mL of each solution was transferred to 125 mL Erlenmeyer flasks, and 0.05 g of the adsorbent was added to each flask. The samples were agitated on an orbital shaker (Nova Ética, model 109-1) at 100 rpm for 2 h at room temperature.

After analysis, a graph was plotted with the ΔpH (pH_initial_ – pH_final_) on the *y*-axis. The pH at the point of zero charge (pH_pzc_) was determined by the intersection of the curve with the *x*-axis, where the ΔpH is equal to zero. Thus obtaining the point of zero charge (PZC), with a strong buffering effect in the region where it is located.^20^

### 2.3 Characterization of Biosorbent

Scanning electron microscopy (SEM), Fourier transform infrared spectroscopy (FT-IR), and X-ray fluorescence (XRF) were used to characterize the stem bark.

SEM was used to analyze the morphological and surface characteristics of the material. Micrographs were obtained using a SU3500 scanning electron microscope (Hitachi, Tokyo, Japan). The samples were mounted on a carbon tape fixed to an aluminum stub and analyzed under low vacuum conditions, with a chamber pressure of 50 Pa and a working distance of 12.1–12.3 mm. The backscattered electron detector (BSE-3D) was employed, with an electron acceleration voltage of 15 kV.

FT-IR analysis was performed using a Cary 630 FTIR spectrometer (Agilent Technologies), equipped with an ATR accessory and operating in the mid-infrared region (4000–650 cm^–1^). Spectra were acquired using Agilent’s Microlab and Resolution Pro software, with a maximum spectral resolution of 2 cm^–1^. Each spectrum represents the average of up to eight scans per wavenumber.

XRF analysis was conducted to identify the chemical elements present in the sample. A Panalytical XRF spectrometer (model Epsilon 1) was used in semiquantitative mode. The material analyzed consisted of loose powder.

### 2.4 Adsorption Thermodynamics

All experiments were carried out at the Analytical and Environmental Chemistry Laboratory (LQAA) of the Regional University of Cariri—URCA, Crato, Ceará, Brazil.

**Table 1:** Critical Comparison between Natural Biosorbents and Conventional Technologies (Nanomaterials, Membranes and Activated Carbon)

technology	selectivity	reuse	cost benefict
biosorbent	high selectivity for certain ions/metals, adjustable by chemical modification	Reusable after simple regeneration (e.g., washing with diluted solutions)	low-cost natural materials, sustainable production
nanomaterials	excellent selectivity, but relies heavily on functionalization	Variable reuse, often limited by agglomeration or degradation	high production cost and complex synthesis
membrane	good physical selectivity (size, load), but limited for complex mixtures	reusable with rigorous operational care	high cost, complex maintenance, foul sensitive
active carbon	limited selectivity, predominant by physical adsorption	reusability possible, but with gradual loss of efficiency	moderate cost, but production may involve nonrenewable resources

The study of thermodynamics in the adsorption process provides insight into the nature of the interaction between the adsorbent and adsorbate and helps optimize operational parameters across different temperatures. In the case of plum shell biosorbents, the thermodynamic parameters analyzed included:

1. Equilibrium constant (*K*_e_): Indicates the extent to which the adsorption process is favored.
2. Enthalpy (Δ*H*°): Reveals whether the process is endothermic or exothermic.
3. Entropy (Δ*S*°): Reflects the change in system disorder during the adsorption process.
4. Gibbs free energy (Δ*G*°): Indicates the spontaneity of the process; negative values confirm that the adsorption is spontaneous.^21^

Equilibrium constants were calculated for each temperature (278.15 K, 298.15 K, and 318.15 K) using eq 1.

$$K^{{}^{\circ}}=\frac{1000\,KeM_{w}[adsorbate{]}^{{}^{\circ}}}{\gamma }$$ 1

where: *K*_e_: equilibrium constant obtained from the best-fit adsorption model. *M*_w_: molecular weight of the adsorbate. [adsorbate]°: standard concentration of adsorbate (1.0 mol L^–1^). γ: activity coefficient (dimensionless).

Using the van’t Hoff equation (eq 2), the standard enthalpy change (Δ*H*°) and standard entropy change (Δ*S*°) were calculated

2 $$\ln K^{{}^{\circ}}=-\frac{\Delta H^{{}^{\circ}}}{R}\frac{1}{T}+\frac{\Delta S^{{}^{\circ}}}{R}$$

where: *R*: universal gas constant (8.314 J mol^–1^ K^–1^); *K*°: adsorption equilibrium constant. *T*: temperature (K).

From the slope and intercept of the linear plot of ln *K*_c_ versus 1/*T*, the values of Δ*H*° and Δ*S*° were determined. Subsequently, the Gibbs free energy change (Δ*G*°) for each temperature was calculated using

3 $$\Delta G^{{}^{\circ}}=\Delta H^{{}^{\circ}}-T\Delta S^{{}^{\circ}}$$

where: Δ*G*°: Gibbs free energy variation (kJ mol^–1^). Δ*H*°: enthalpy variation (adsorption heat) (kJ mol^–1^). *T*: temperature (K), Δ*S*°: entropy variation (kJ mol^–1^ K^–1^).

### 2.5 Batch Kinetic Adsorption

Batch adsorption tests were performed using plum stem bark as the adsorbent to obtain kinetic and equilibrium data, evaluate theoretical models, and interpret the associated parameters. The experiments were conducted at different contact times: 1, 2, 4, 8, 16, 32, 60, and 120 min, with the temperature maintained at 298.15 K. All measurements were carried out in triplicate using 125 mL Erlenmeyer flasks.

The buffer solution used consisted of acetic acid and sodium acetate, adjusted to pH 5.5. According to Ferenj et al.,^22^ aqueous solutions with higher pH values promote deprotonation of the adsorbent surface, which enhances the electrostatic attraction between the more negatively charged adsorbent surface and Pb­(II) ions, thereby increasing the adsorption capacity. However, a significant decrease in Pb­(II) adsorption at pH values between 8 and 10 can be attributed to the reduced availability of free Pb­(II) ions due to the precipitation of lead hydroxide [Pb­(OH)_2_]. Figure 1 illustrates the adsorption process over time.

**Figure 1:**
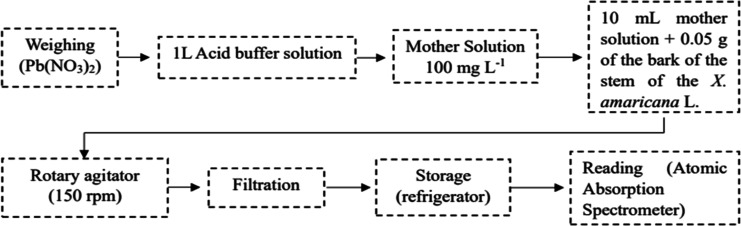
Schematic representation of the adsorption kinetics process.

The samples were analyzed using a Varian flame atomic absorption spectrometer (FAAS), model SpectrAA 50B. FAAS was employed to determine the metal concentrations in the samples by measuring the amount of light absorbed by metal atoms vaporized in a flame.

The obtained concentrations were then used to calculate the adsorption capacities (*q*) according to eq 4

4 $$q=\frac{\left(C_{0}-C_{f}\right)V}{m}$$

where: *q*: amount of metal adsorbed per unit mass of adsorbent (mg g^–1^), *C*_0_: initial concentration of metal ions in solution (mg L^–1^), *C*_f_: equilibrium concentration of metal ions in solution (mg L^–1^), *V* = volume of the solution (L).

The values obtained for *q* were essential for evaluating the fit of theoretical kinetic models to the experimental data, and subsequently, for analyzing and discussing the adsorption parameters and possible mechanisms involved. The nonlinear kinetic models evaluated included the pseudo-first order (PFO) and pseudo-second order (PSO) models. Additionally, to understand the diffusion mechanisms involved in the adsorption process, the Boyd model was applied, allowing the identification of the controlling steps of the adsorption kinetics.^23^

A summary of the models and their respective equations is presented in Table 2.

**Table 2:** Non-linear Equations and Kinetic Model Parameters

kinetic models	nonlinear equations	parameters
pseudo-first order (PFO)^24,25^	*q*_t_ = *q*_e_ × (1 – *e* ^–*k*1×*t*^)	*q*_t_: adsorption capacity at time *t* (mg g^–1^)
		*q*_e_: adsorption capacity at equilibrium (mg g^–1^)
		*k*_1_: pseudo-first order adsorption rate constant order (min^–1^)
		*t*: time (min)
pseudo-second order (PSO)^26^	$q_{t}=\frac{{q_{e}}^{2}\times k_{2}\times t}{1+q_{e}\times k_{2}\times t}$	*k*_2_: pseudo-second order adsorption rate constant order (g mg^–1^ min^–1^)
Boyd (intrapore diffusion model)^27,28^	If *F* < 0.85 $t=({\sqrt{\pi }-\sqrt{(\pi -(\frac{\pi^{2}F}{3}}))}^{2}$	*F*: *q*_t_/*q*_e_
	If *F* > 0.85	*B*_t_: Math function for *F*
	*Bt* = −0.49770 – ln(1 *F*)	*D*: coefficient for effective diffusion (cm^2^ min^–1^) *r*: particle radius (cm)
	$F=\frac{q_{t}}{q_{e}},B=\frac{\pi^{2}D}{r^{2}}$	*B*_B_: Boyd model constant

### 2.6 Batch Adsorption Isotherms

Adsorption isotherms are thermodynamic models that describe the relationship between the amount of adsorbate retained on the surface of the adsorbent and its concentration in solution at a constant temperature, after the system has reached equilibrium.

In this study, isotherm experiments were conducted over a contact time of 20 min, with the temperature controlled at 278.15, 298.15, and 318.15 K, respectively. These conditions allowed for the evaluation of how temperature influences the adsorption capacity and affinity between the adsorbent and Pb^2+^ ions.

Figure 2 illustrates the experimental procedure used for generating the isotherm data.

**Figure 2:**
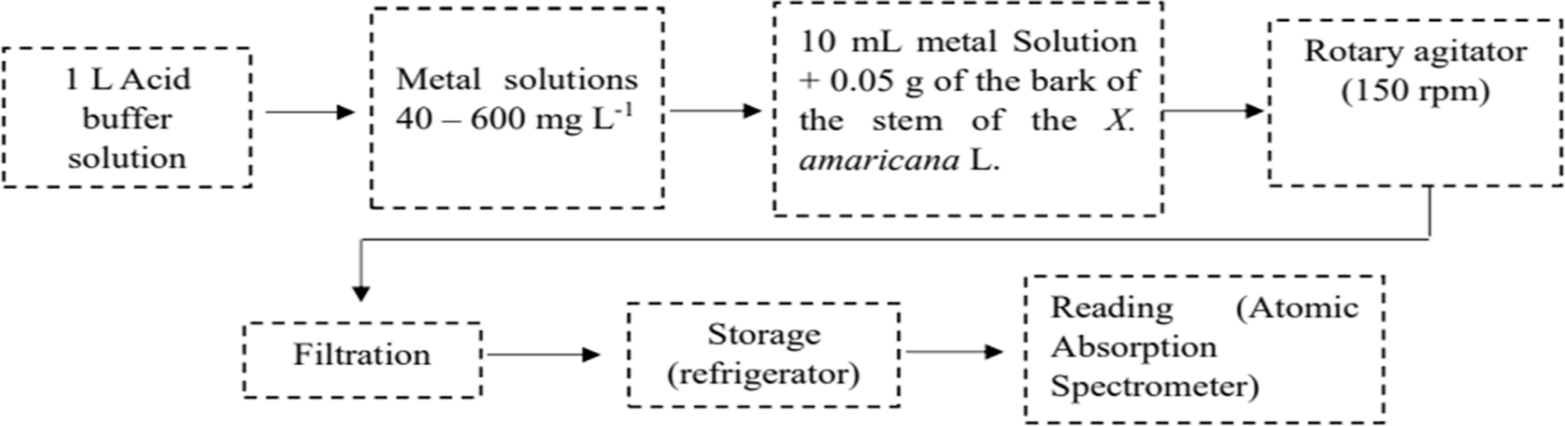
Diagram of the adsorption isotherm process.

The Langmuir, Temkin II, Sips, and BET nonlinear models were applied using the equations described in Table 3 to determine which temperature favored adsorption and to identify the model that best represented the adsorption process.

**Table 3:** Non-linear Adsorption Isotherm Models and Parameters

isotherm	nonlinear equations	parameters
Langmuir^29^	$q_{e}=\frac{q_{\max}\times K_{L}\times C_{e}}{1+K_{L}\times C_{e}}$	*q*_e_: adsorption capacity at equilibrium (mg g^–1^)
		*q*_max_: maximum adsorption capacity (mg g^–1^)
		*K*_L_: Langmuir constante (L mg^–1^)
		*C*_e_: final concentration of the ion in solution (mg L^–1^)
Temkin II^30^	*q* = *qT* × ln(1 + *K*_T_ × Cf)	*q*_T_: adsorption capacity (mg g^–1^)
		*K*_T_: Temkin constant (L mg^–1^)
		*C*_e_: final concentration of the ion in solution (mg L^–1^)
Sips^31^	$q_{e}=\frac{q_{\max}\times {K_{s}\times C_{e}}^{\beta_{s}}}{1+{K_{s}\times C_{e}}^{\beta_{s}}}$	*q*_e_: adsorption capacity at equilibrium (mg g^–1^)
		*q*_max_: maximum adsorption capacity (mg g^–1^)
		*K*_s_: sips constant (L mg^–1^)
		final concentration of the ion in solution (mg L^–1^)
		β_s_: Sips exponent (dimensionless)
BET^32^	$q=\left[\frac{q_{\max}b_{1}C_{e}}{1+\left(b_{1}-b_{2}\right)C_{e}}\right]\left[\frac{1}{1-b_{2}C_{e}}\right]$	*q*_max:_ maximum adsorption capacity (mg g^–1^)
		*C*_e_: final concentration of the ion in solution (mg L^–1^) *b*_1_: equilibrium constant (L mg^–1^)
		*b*_2_: equilibrium constant (L mg^–1^)
		α (—): BET constant related to heat of adsorption and heat of vapor liquefaction.

### 2.7 Effect of Sorbent Dosage

The determination of the optimal adsorbent dosage in an adsorption system is fundamental for evaluating the financial cost of the process and, consequently, for assessing the economic feasibility of implementing a given treatment method.^33^

For this purpose, aliquots of 10 mL of Pb­(II) solution (≈100 mg L^–1^) were added to varying masses of biosorbent to obtain dosages ranging from 1 to 10 g L^–1^. The experiments were performed under the following operational conditions: pH 5.5, temperature 298.15 K, contact time 60 min, and agitation speed 100 rpm.

### 2.8 Desorption and Reusability Tests

The desorption experiments were performed according to the methodology reported elsewhere,^34^ using *X. americana* bark as the sorbent after the completion of the Pb­(II) adsorption tests. The biosorbent was first dried in an oven at 315.15 K for 20 min, and then mixed with 50 mL of 0.1 M HCl solution (in quintuplicate) under stirring at 100 rpm and 298.15 K. At predetermined time intervals, the liquid phase was collected, filtered, and the concentration of Pb­(II) ions was measured using flame atomic absorption spectroscopy (FAAS). The desorption efficiency (DE) was calculated using eq 5,^34,35^ as follows

5 $$DE\;\left(\%\right)=\frac{C_{t}\times V}{m0}\times 100$$

where *C*_t_(mg·L^–1^) is the concentration of copper ions in the desorption solution at time *t* (min), *V* is volume of the desorption solution, and *m*_0_ (g) is the amount of Pb­(II) ions sorbed in the sorption test.

For the reusability tests, a selected sample of the sorbent underwent three consecutive sorption/desorption cycles under the conditions previously described.

### 2.9 Effect of Coexisting Ions on Lead Sorption

The influence of coexisting ions was investigated by comparing the sorption performance under three different conditions: (a) Pb­(II) alone; (b) Pb­(II) + 0.01 M KCl; and (c) Pb­(II) + 0.01 M CaCl_2_. The other operational conditions were as follows: sorbent dosage = 5 g L^–1^; initial Pb­(II) concentration ≈ 100 mg L^–1^; buffered pH = 5.5; contact time = 60 min; agitation speed = 100 rpm; temperature = 298.15 K.

## 3 Results and Discussions

### 3.1 pH_pzc_

The analysis of the point of zero charge (pH_pzc_) is essential for identifying the pH at which the surface charge of the adsorbent becomes neutral, meaning there is an equal balance between positive and negative charges. This allows for the prediction of the surface charge behavior: the surface becomes positively charged when pH < pH_pzc_ and negatively charged when pH > pH_pzc_.^36^ positive less than pzc, net higher than pzc.

The results obtained (Figure 3) indicate that the pH_pzc_ of the adsorbent is 6.0. Therefore, within the pH range of 5.5–6.0, the surface of the adsorbent likely exhibits a net negative charge, favoring the adsorption of negative charged species such as Pb­(II) ions. Conversely, at pH values above the pH_pzc_, the adsorption of negatively charged species may be less favorable due to increased electrostatic repulsion.

**Figure 3:**
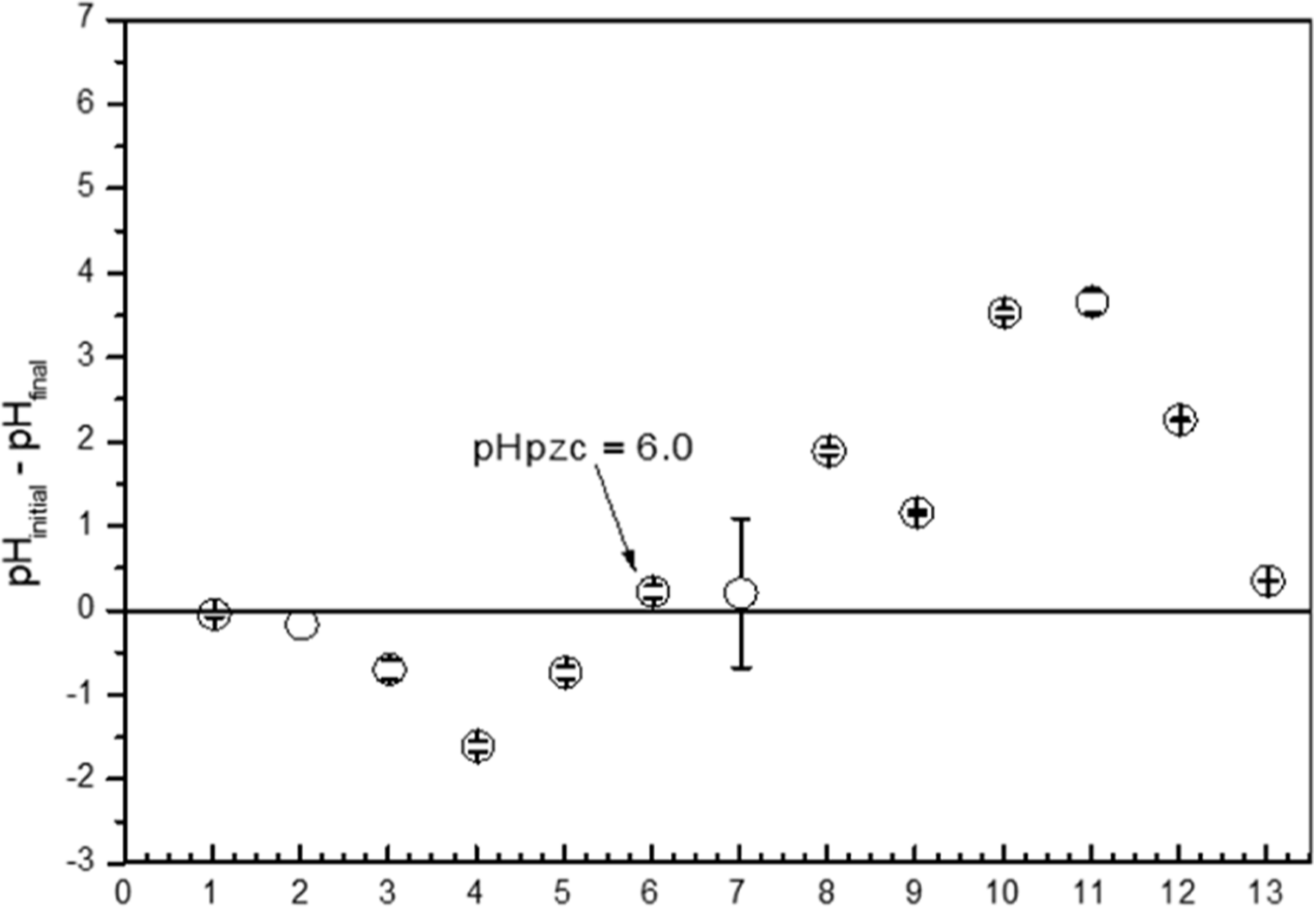
Adsorption capacity of Pb^2+^ by *Ximenia americana* stem bark as a function of pH, using 10 mL of solution, 5 g L^–1^ of adsorbent dosage, and a contact time of 120 min.

### 3.2 Scanning Electron Microscopy

Figure 4a,b present the SEM micrographs, which reveal the structural morphology of the plum stem bark.

**Figure 4:**
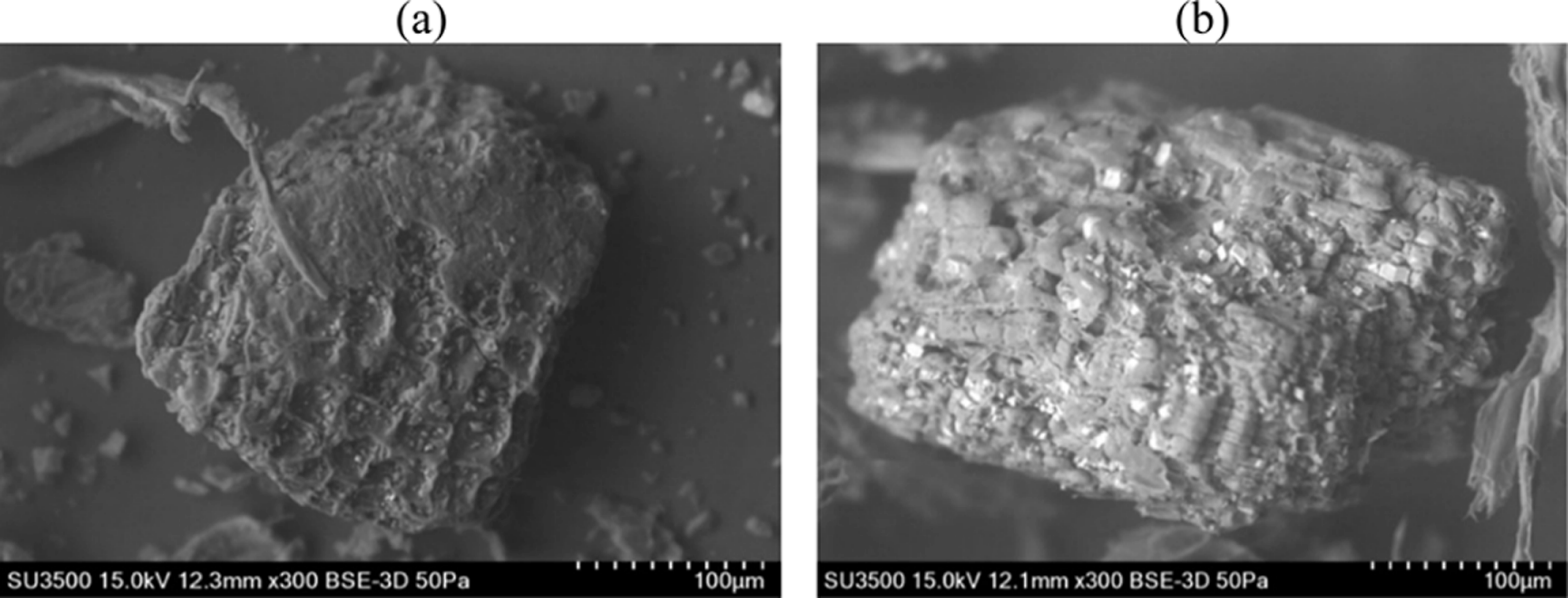
SEM images of *Ximenia americana* L. stem bark: (a) before Pb^2+^ adsorption and (b) after Pb^2+^ adsorption, captured at 300× magnification.

According to Costa et al.,^37^ porous solids are among the most efficient materials for adsorption processes due to their high surface area.

Figure 4a shows that the surface of the sample has an irregular and rough texture, with various formations and structures, indicating that the sample has been processed, The presence of empty spaces suggests good permeability of the material. Meanwhile, Figure 4b presents an image with an irregular and rigid structure, featuring a rough and surface with a variety of shapes and sizes, which may suggest a mixture of materials or the presence of agglomerates. Some particles exhibit varying shapes, and certain areas appear to have a crystalline structure, possibly indicating the presence of minerals ormetallic compounds, as further detailed in Table 4. Other regions show smaller, more irregular particles that vary in size.

**Table 4:** Fluorescence Spectra of *Ximenia americana* L. Stem Bark before and after Adsorption of Pb­(II) Ions

compound	concentration in plum peel (*in natura*) (%)	concentration in plum peel treated with Pb^2+^ ions (%)
Si	0.369	0.456
Cl	0.488	0.375
K	2.012	0.466
Ca	16.19	18.36
Fe	0.245	0.195
Pb	0	13.12
Ge	0.281	0.199
Sr	0.323	0.278
Zr	0.224	0
Ba	0.181	0.193
Yb	0.422	0.581
Lu	0.342	0.280

The results obtained by Abdelwahab and Gasser et al.^38,39^ demonstrate cracks and pores of different sizes and shapes on the external surface of adsorbents used for lead ion removal, exhibiting behavior similar to that observed in this study. The heterogeneous structure of the adsorbent favored adsorption, enabling efficient fixation of metal ions.

### 3.3 Fourier Transform Infrared Spectroscopy (FT-IR)

According to the FTIR analysis, Figure 5 shows the transmittance (*T*) as a percentage in relation to the wavenumber (cm^–1^). The vertical axis represents the transmittance (ranging from 78% to 100%), while the horizontal axis represents the wavenumbers (from 4000 to 1000 cm^–1^).

**Figure 5:**
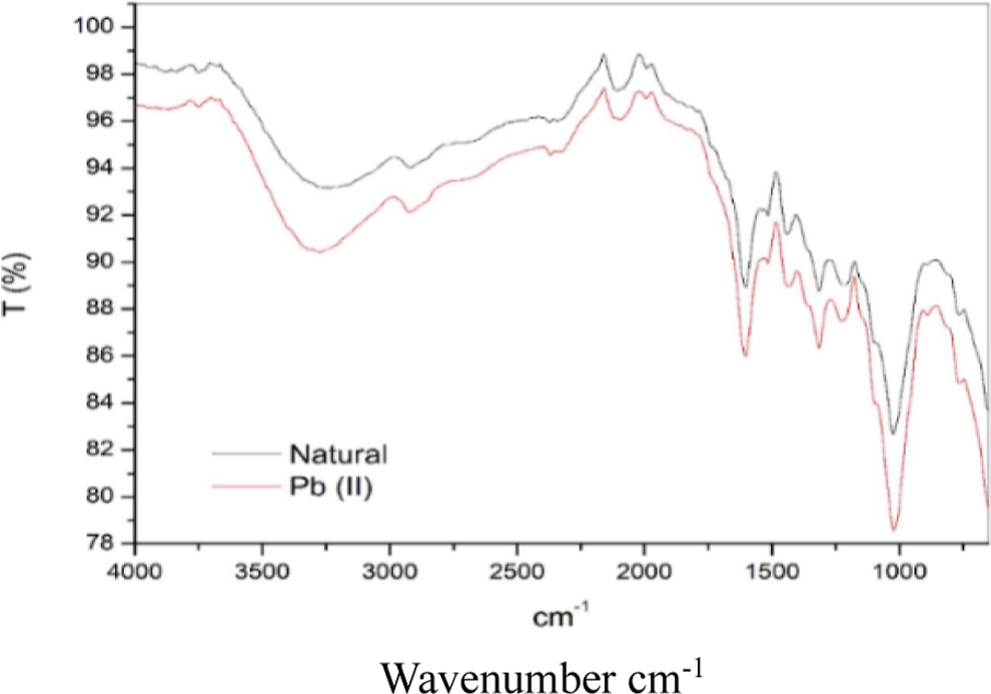
Fourier transform infrared spectroscopy (FT-IR) spectra of *Ximenia americana* L. bark in natura and after treatment with Pb^2+^ ions.

The differences observed in the spectra may indicate how the presence of Pb­(II) influences the chemical properties of the sample. It is possible to observe changes in the bands of the raw sample compared to the metal-treated one. The relevant bands are: 3700–3200 cm^–1^ (O–H bond stretching vibrations—alcohols, phenols); 3300–2500 cm^–1^ (N–H bond stretching vibrations—amines); 3000–2800 cm^–1^ (C–H stretching vibrations—hydrocarbons, alcohols, and carboxylic acids); 1750–1600 cm^–1^ (C=O stretching vibrations, characteristic of carbonyls, esters, and carboxylic acids);^40^ 1600–1500 cm^–1^ (C=C bond deformation vibrations – aromatic compounds); 1500–1300 cm^–1^ (C–H bond deformation vibrations—aromatic compounds); 1300–1000 cm^–1^ (C–O stretching vibrations—alcohols and esters).^41^

The band around 1000 cm^–1^ is commonly attributed to the stretching vibrations of the C–O bonds present in alcohols, ethers, and esters, indicating the presence of hydroxylated functional groups on the material surface^42^ Silverstein et al.,.^43^ and Tavares et al.,^42^ using coffee husk, identified bands at 1022 cm^–1^, attributed to the vibrational stretching of C–O. The 1200–1000 cm^–1^ range corresponds to stretching vibrations for C–O and C–N bonds, important for identifying sugars, polysaccharides, and amines. The 1000–600 cm^–1^ region involves more complex deformation vibrations, often related to the molecular skeleton of inorganic or organic compounds, including bonds such as Si–O, P–O, or C–H.

Studies by Lima^44^ indicate that functional groups such as carbonyls on the adsorbent surface can significantly influence heavy metal adsorption by favoring complex formation with metal ions. Functional groups such as esters, although less reactive than carboxyls or hydroxyls, can contribute to metal ion adsorption in composite materials by enhancing system stability or facilitating intermolecular interactions.^45^

In the study by Carneiro et al.,^46^ the ethanolic extract of *X. americana* L. stem bark showed significant concentrations of phytoconstituents such as catechin, (−)-epicatechin, rutin, and myricetin, which contain −OH bonds of alcohols and phenols, as confirmed by FTIR analysis (Figure 5). Given that the percentage transmittance of the band at 3700–3200 cm^–1^ decreases after adsorption with Pb^2+^ ions, it is suggested that these functional groups are directly involved in the adsorption process.

### 3.4 X-ray Fluorescence (XRF)

XRF results are shown in Table 4. In the fresh plum sample, silicon (Si) and potassium (K) are present in significant quantities (0.36% and 2.01%, respectively). The concentration of lead (Pb) is 0%, indicating the absence of this element in the natural sample, while the sample treated with Pb^2+^ ions has a notable concentration of various elements, such as potassium (0.46%) and calcium (18.36%). The concentration of lead (Pb) rises to 13.12%, suggesting that treatment with plum ions may influence the absorption or mobilization of elements in the plum skin.

### 3.5 Kinetic Equilibrium Studies

The rate at which the solute is removed varies according to its physical and chemical characteristics. When evaluating the influence of contact time on the adsorption process, it was found that plum bark was effective in removing lead ions (Figure 6). In a short time, high percentages of metal removal were obtained, reaching equilibrium in 20 min. The equilibrium concentration obtained experimentally when using the adsorbent was close to 15.5 mg g^–1^.

**Figure 6:**
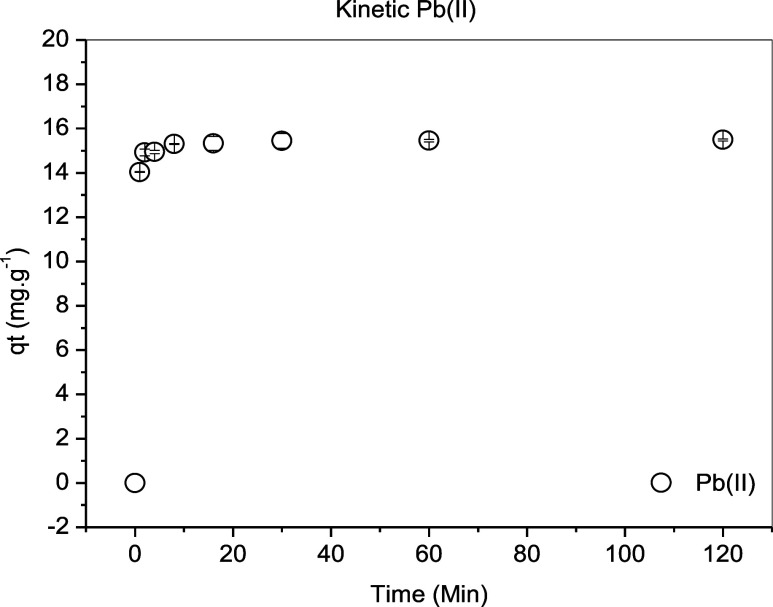
Effect of contact time on the removal of Pb^2+^ ions using the stem bark of *Ximenia americana* L.

The data sets were fitted to the pseudo-first, pseudo-second order models and the Boyd diffusion model, as shown in Figure 7. The parameters associated with the fits are presented in Table 5.

**Figure 7:**
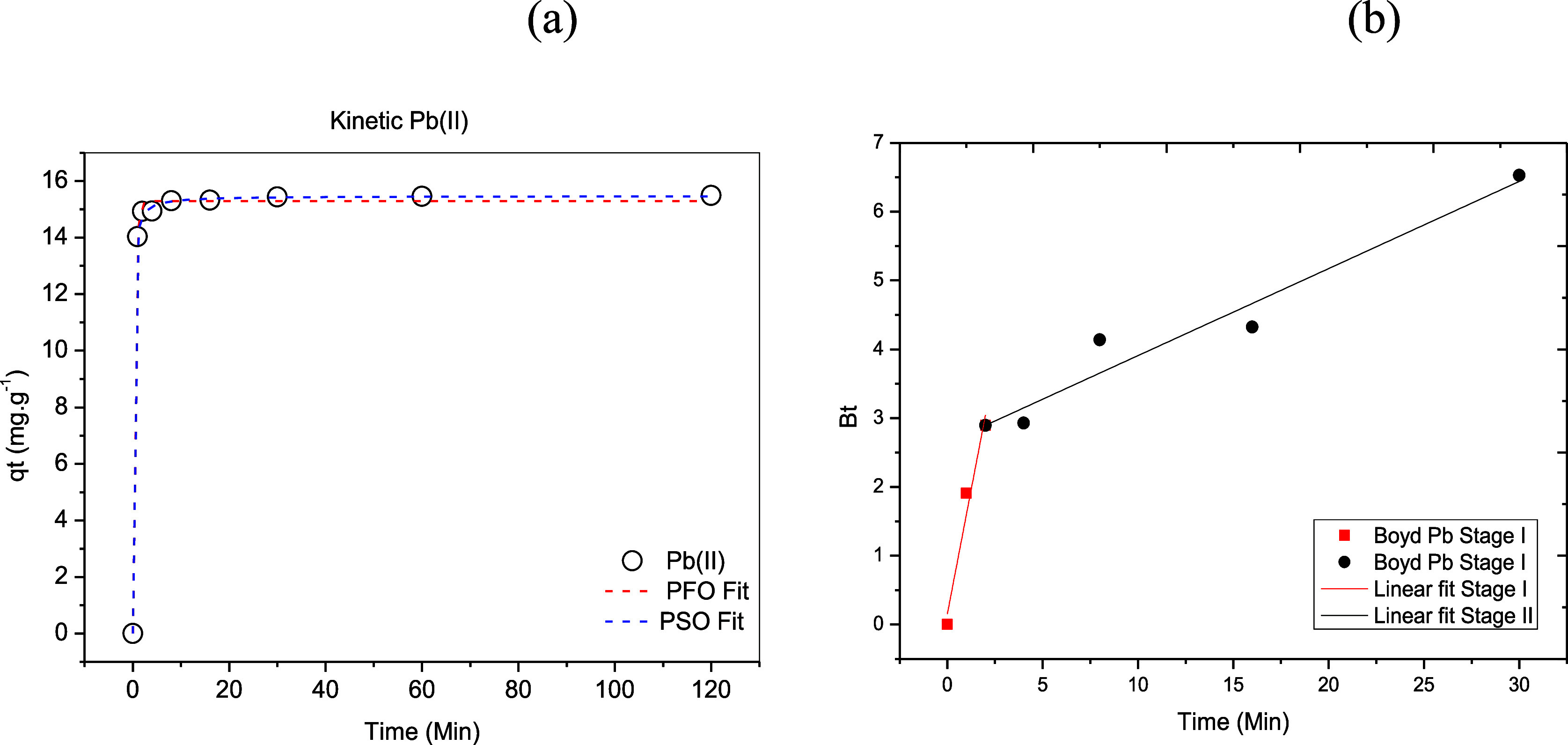
Kinetic study of Pb^2+^ ion adsorption onto *Ximenia americana* L. stem bark: (a) Langergren (pseudo-first order) and Ho (pseudo-second order) kinetic models; (b) Boyd diffusion model.

**Table 5:** Kinetic Parameters for the Adsorption of Pb^2+^ Ions Onto *Ximenia americana* L. Stem Bark, Fitted Using the Lagergren (Pseudo-first Order), Ho (Pseudo-second Order) Models, and Boyd Diffusion Model

pseudo-first order—PFO nonlinear model						
metal	*q*_e_ (mg g^–1^)	adjusted *R* ^2^	SSE	χ^2^	*k*_1_ (min^–1^)	
Pb(II)	15.29	0.998	0.279	0.039	2.464	

pseudo-segund order—PSO nonlinear model					
metal	*q*_e_ (mg g^–1^)	adjusted *R* ^2^	SSE	χ^2^	*k*_2_ (g mg^–1^ min^–1^)
Pb(II)	15.47	0.999	0.0637	0.0091	0.658

Boyd model					
metal	*B*	*D* (cm^2^ min)	intercept (*b*)	adjusted *R* ^2^	SSE
Pb(II) stage I	1.446	1.143 × 10^–7^	0.153	0.935	0.141
Pb(II) stage II	0.127	1.001 × 10^–8^	2.641	0.938	0.407

The coefficients of determination (*R* ^2^) shown in Table 5 indicate that the pseudo-second order model (*R* ^2^ = 0.999) (Figure 7a) fits the experimental kinetic data better than the pseudo-first order model (*R* ^2^ = 0.998). Additionally, the equilibrium capacity estimated using the pseudo-second order model, 15.47 mg g^–1^, is closer to the experimentally obtained value than that estimated by the pseudo-first order model. The pseudo-second order rate expression is commonly used to describe chemisorption involving valence forces, through the sharing or exchange of electrons between the adsorbent and the adsorbate, such as covalent bonding and ion exchange. The advantage of fitting this model is that it estimates the equilibrium adsorption capacity of the adsorbent material.^47^

Other authors have used fruit or plant peels, such as Silva et al.,^48^ who studied lead ion adsorption using banana peel; Batista et al.^49^ using mandarin peel; Lima et al.^50^ using mandarin peel and sugar cane bagasse; and E da Siva et al.^51^ who chemically modified coconut shell. They found that the pseudo-second order model fitted best. The presence of carboxylic groups and amines in these materials, as well as in the plum shell, can facilitate complex formation with Pb^2+^ ions.

Regarding the Boyd model, Figure 7b and the results in Table 5 show that the adsorption process is controlled by intraparticle diffusion at the initial stages, as the plot passes through the origin with an intersection value of 0.153. At this point, the film has not yet formed, so particle transit occurs without interference from it.

### 3.6 Adsorption Isotherm Studies

Adsorption isotherms represent the relationship between the quantity of substance adsorbed (*q*) and the equilibrium concentration of the substance (*C*_e_) at a constant temperature (Figure 8). These isotherms are fundamental for elucidating both the practical aspects of adsorption in industrial applications, such as purification and separation processes, and the thermodynamic and morphological properties of the adsorbent material, including its structural characteristics and adsorption capacity. Consequently, adsorption isotherms play a crucial role in optimizing adsorption systems and enhancing the understanding of the underlying mechanisms governing the adsorption process.

**Figure 8:**
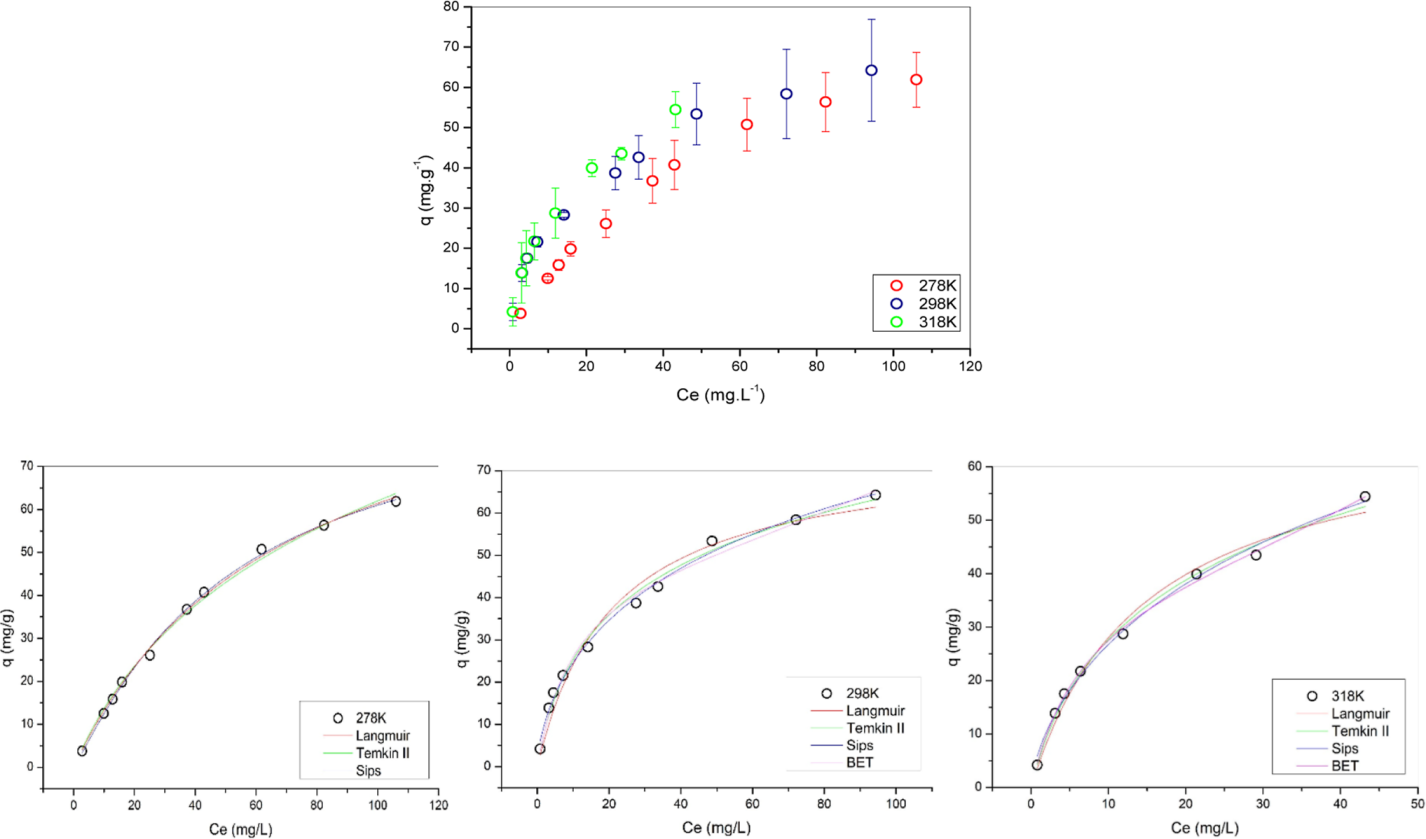
Associated errors and experimental results of the Langmuir, Temkin II, Sips and BET adsorption equilibrium models for Pb^2+^ ions at 278.15 K, 298.15 and 318.15 K.

Determining the equilibrium time is important because it is a fundamental parameter for verifying the efficiency of adsorption systems and obtaining adsorption isotherms.^52^ The Langmuir, Temkin II, Sips and BET nonlinear models were used to study the equilibrium of the Pb­(II) ion adsorption system. Figure 8 and Table 7 represent the data.

The Sips model showed the best fit for the adsorption of Pb^2+^ ions, with the highest adjusted *R* ^2^ value (0.998) at 278.15 K, as well as showing the lowest SSE and χ^2^ values at temperatures of 278.15 and 318.15 K. This reinforces the idea that adsorption occurs in a heterogeneous manner, with different types of active sites on the surface of the adsorbent indicating an excellent performance in terms of fit. The Sips model, although empirical like the Freundlich model, is mathematically similar to the Langmuir model, but includes an additional parameter, βs.

Langmuir and BET *q* have similar values at 278.15 K, indicating that both capture the maximum capacity well. However, the Langmuir model has a sharper decrease at higher temperatures, while the Sips model shows an unexpected increase at 298.15 K, the BET model has good *q*_max_ results but has high errors making it inferior to the Sips model, at higher temperatures the good fit of the BET^33^ indicates that the adsorption process may be occurring in a multilayer fashion, rather than just a monolayer as predicted by the Langmuir model.

While the Temkin II model has significantly lower maximum capacities, The affinity constant *K*_L_ in the Langmuir model shows an increase with temperature, indicating greater affinity for adsorption as the temperature increases, while the *K*_S_ in the Sips and Temkin II models also increases with temperature, confirming the idea that adsorption energy favors higher temperatures. Thus, the Sips model stands out as the most robust and appropriate for describing the adsorption of Pb^2+^ ions, providing a better representation of the experimental data due to its superior statistical fit and understanding of molecular interactions.

Other authors such as Feitosa et al.,^53^ Santos et al.^35^ used biosorbent materials such as barley dust (*Hordeum vulgare* L.) and the modified peels of *Ziziphus joazeiro* and obtained maximum adsorption capacity values of 25.76 mg g^–1^ and 62.5 mg g^–1^ and *q*_max_ (adjusted) of 0.9932 and 0.983 respectively, thus demonstrating that bioadsorbent materials have a high adsorption capacity when using the powder *in natura* or modified. Table 6 expands the comparisons made previously, bringing complementary data.

**Table 6:** Comparison of Plant Biosorbents for Pb^2+^ Removal in Aqueous Solutions

vegetable material	*q*_max_ (mg g^–1^)	dominant isotherm	Kinetic model	pretreatment	reuse	selectivy of (Pb^2+^)	origin and cost	reference
Ximenia americana L. (natural bark)	138.20	Sips	pseudo-second order	no	yes	loud	regional abundante/low	present study
Senna Tora pods	0.51	Langmuir	Pseudo-first order	yes	not tested	Media	tropical and subtropical regions/low	Silas and Osagie^54^
Anacardium occidentale	8734	Langmuir	Pseudo-second order	yes	yes	not tested	agricultural byproduct/Low	Nuithitikul et al.^55^
Mangifera indica L. (caro)	78.54	Freundlich	Pseudo-second order	yes	yes	not tested	abundant/very low	Raasch et al.^56^
Coffea arábica (bark)	37.04	Langmuir	Pseudo-second order	no	Not tested	Not tested	abundant/very low	Alhogbi^57^

Determining the equilibrium time is crucial, as it is a fundamental parameter for assessing the efficiency of adsorption systems and for obtaining adsorption isotherms.^52^ The Langmuir, Temkin II, Sips, and BET nonlinear models were employed to study the equilibrium behavior of the Pb­(II) ion adsorption system. The results are presented in Figure 8 and Table 6.

**Table 7:** Adsorption Isotherms for Pb^2+^ Ion Removal Using the Stem Bark of *Ximenia americana* L.: Langmuir, Temkin II, Sips, and BET Models

Langmuir			
parameter/Temp (K)	278.15	298.15	318.15
*q*_max_ (mg g^–1^)	103.4	74.97	68.88
*K*_L_ (L mg^–1^)	0.015	0.047	0.068
adjusted *R* ^2^	0.997	0.975	0.983
SSE	8.023	81.69	28.04
χ^2^	1.003	10.21	4.673
*W*_i_ (%)	67.10	0.300	0.800
Temkin II			
*K*_T_ (L mg^–1^)	0.046	0.273	0.299
*Q*_t_ (mg g^–1^)	35.80	19.21	19.96
adjusted *R* ^2^	0.995	0.991	0.993
SSE	15.96	28.53	11.28
χ^2^	1.995	3.566	1.881
*W*_i_ (%)	2.2	51.4	31.2
Sips			
*q*_max_ (mg g^–1^)	90.73	138.2	133.5
*K*_S_ (L mg^–1^)	0.012	0.051	0.053
βs	1.109	0.621	0.671
adjusted *R* ^2^	0.998	0.994	0.994
SSE	5.500	16.58	9.190
χ^2^	0.785	2.369	1.832
*W*_i_ (%)	26.7	46.6	2.1
BET			
*q*_max_ (mg g^–1^)	103.4	42.21	40.64
*K*_S_ (L mg^–1^)	0.014	0.1015	0.154
*W*	0	0.00316	0,00745
adjusted *R* ^2^	0.997	0.988	0.997
SSE	8.023	32.32	3.875
χ^2^	1.146	4.617	0.775
*W*_i_ (%)	4.0	1.7	65.9

The Sips model provided the best fit for Pb^2+^ ion adsorption, exhibiting the highest adjusted *R* ^2^ value (0.998) at 278.15 K, along with the lowest SSE and χ^2^ values at temperatures of 278.15 and 318.15 K. This finding supports the notion that adsorption occurs heterogeneously, involving various types of active sites on the adsorbent surface, and demonstrates excellent fitting performance. Although empirical like the Freundlich model, the Sips model is mathematically similar to the Langmuir model but incorporates an additional parameter, β*s*.

At 278.15 K, the Langmuir and BET models yielded similar maximum adsorption capacities (*q*_max_), indicating that both effectively capture the adsorbent’s maximum capacity. However, the Langmuir model shows a more pronounced decrease in capacity at higher temperatures, whereas the Sips model displays an unexpected increase at 298.15 K. Although the BET model achieves good *q*_max_ values, its higher errors render it less accurate than the Sips model. At elevated temperatures, the good fit of the BET model suggests that the adsorption process may involve multilayer adsorption rather than the monolayer adsorption predicted by the Langmuir model.

The Temkin II model, in contrast, predicts significantly lower maximum adsorption capacities. The affinity constant (*K*_L_) in the Langmuir model increases with temperature, indicating enhanced adsorption affinity at higher temperatures. Similarly, the constants *K*_S_ in the Sips and Temkin II models also increase with temperature, confirming that adsorption is energetically favored by rising temperatures.

Therefore, the Sips model stands out as the most robust and appropriate for describing Pb^2+^ ion adsorption, offering a superior representation of the experimental data due to its better statistical fit and its ability to capture the molecular interactions involved.

Similar studies by Feitosa et al.^53^ and Santos et al.^35^ used bioadsorbent materials such as barley dust (*H. vulgare* L.) and modified peels of *Z. joazeiro*, achieving maximum adsorption capacities of 25.76 mg g^–1^ and 62.5 mg g^–1^, respectively, with adjusted *R* ^2^ values of 0.9932 and 0.983. These results further demonstrate the high adsorption capacities of bioadsorbent materials, whether used in their natural or modified forms.

### 3.7 Thermodynamic Studies

The thermodynamic parameters for the adsorption of Pb^2+^ ions, presented in Table 8, were calculated based on eqs 1–3. The parameters Gibbs free energy (Δ*G*°), enthalpy (Δ*H*°), and entropy (Δ*S*°)­are essential for understanding the spontaneity and nature of the adsorption process. Δ*G*° indicates whether the process is spontaneous, Δ*H*° provides insight into the heat exchange involved, and Δ*S*° reflects changes in the disorder of the system (see Table 8).

**Table 8:** Thermodynamic Parameters for the Adsorption of Pb^2+^ Ions Using the Stem Bark of *Ximenia americana* L

temperature (K)	Δ*G*° (kJ mol^–1^)		Δ*H*° (kJ mol^–1^)	Δ*S*° (kJ mol^–1^ K^–1^)
278.15	–3.21	±3.73	77.4 ± 21.5	0.29 ± 0.0639
298.15	–9.01	±2.45		
318.15	–14.8	±1.17		

The variation in Gibbs free energy indicates the spontaneity of the reaction. The negative values of −3.21, −9.01, and −14.8 kJ mol^–1^ demonstrate that the reaction is spontaneous. Moreover, as the temperature increases, these values become more negative, suggesting that adsorption is more favorable at higher temperatures.

Enthalpy represents the amount of heat absorbed or released during the reaction. The positive value of 77.4 kJ mol^–1^ indicates that the adsorption process is endothermic. This implies that increasing the temperature may enhance the adsorption capacity.

The entropy value of 0.29 kJ mol^–1^ K^–1^ suggests that the adsorption process leads to an increase in the disorder of the system, which is characteristic of many adsorption phenomena where molecular organization becomes more random.

### 3.8 Effect of Sorbent Dosage

Figure 9 illustrates the influence of adsorbent dosage on the adsorption efficiency of Pb^2+^. Aliquots of 10 mL of Pb^2+^ solution (100 mg L^–1^) were added to varying masses of the adsorbent to achieve dosages of 1 g L^–1^, 2.5 g L^–1^, 5 g L^–1^, 7.5 g L^–1^, and 10 g L^–1^.

**Figure 9:**
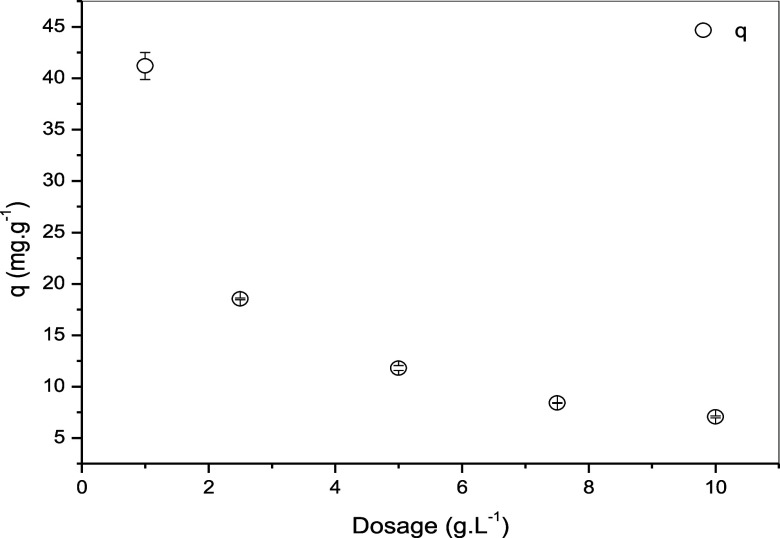
Effect of the variation of the adsorbent dose mass/liquid ratios: 0.01–10 g L^–1^; initial concentration of Pb^2+^: 0.1 mol L; agitation speed 100 rpm; volume of solution: 10 mL; temperature 298.15 K; Initial pH 5.5; contact time: 60 min.

This variation in adsorbent dosage allows for the evaluation of how the amount of adsorbent affects the adsorption process.

Lead removal efficiency increases as the amount of adsorbent increases, reaching a maximum value of 78.68% at an adsorbent mass of 0.1 g. This improvement is attributed to the greater availability of surface area and adsorption sites. However, it is noteworthy that even lower adsorbent dosages achieve satisfactory adsorption levels, indicating that the adsorbent mass does not solely determine the adsorption efficiency. The process is influenced by multiple factors, including the solution pH, the surface area and porosity of the adsorbent, and the presence of active functional groups responsible for binding the metal ions.

### 3.9 Reusability Tests

To assess the reusability of the adsorbent, three adsorption–desorption cycles were conducted under the experimental conditions summarized in Figure 10. As observed, the removal efficiency decreased with each successive cycle; however, the reduction was not substantial. After the second use, the adsorbent’s capacity decreased by 23.0%, and following the third cycle, the reduction reached 27.4%. These results indicate that the adsorbent can be reused effectively, as its adsorption capacity does not undergo drastic deterioration over multiple cycles.

**Figure 10:**
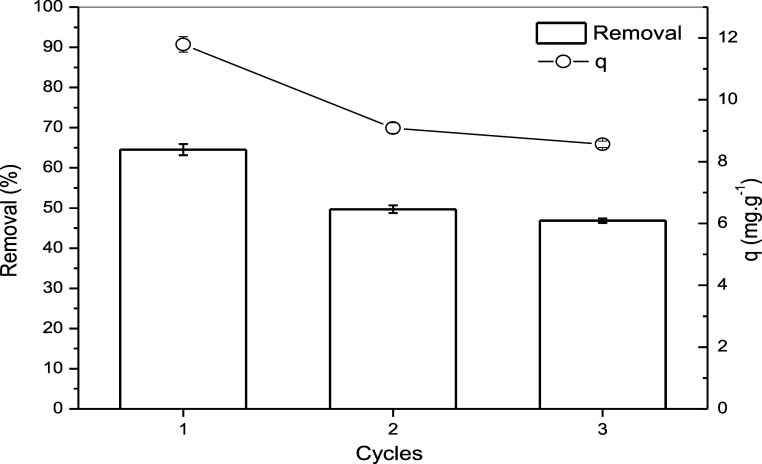
Effect of the adsorbent dosage on the sorption of lead. Test conditions: initial concentration of Pb^2+^: 0.1 mol L; agitation speed 100 rpm; volume of solution: 10 mL; mass of sorbent = 0.05 g temperature 298.15 K; pH 5.5; contact time: 60 min.

### 3.10 Effect of Coexisting Ions

According to studies conducted by Macedo et al.,^58^ naturally occurring ions in water, such as potassium (K^+^) and calcium (Ca^2+^), can compete with lead ions (Pb^2+^) for active adsorption sites, potentially decreasing the efficiency of the metal removal process. This competition occurs because K^+^ and Ca^2+^ ions have charges and ionic sizes similar to those of Pb^2+^, which affects the availability of adsorption sites for lead. The effect of potassium and calcium ions on lead adsorption is shown in Figure 11. The results indicate that these ions do not appear to compete significantly for the same active sites, as lead removal efficiency remains close to 90%.

**Figure 11:**
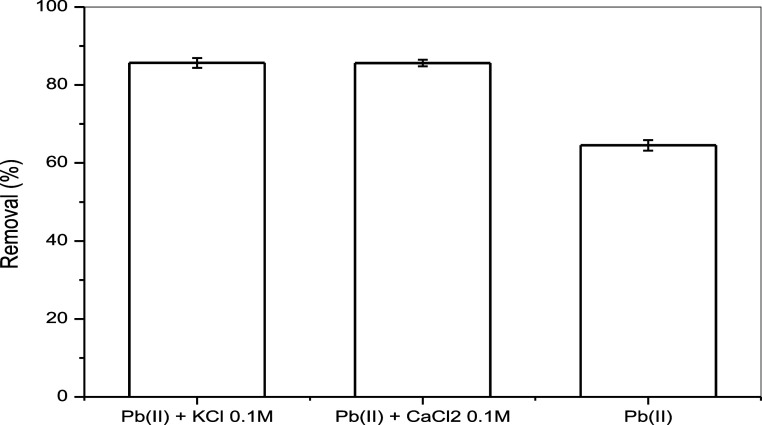
Effect of the presence of potassium and calcium ions on the sorption of lead. Test conditions: initial concentration of Pb^2+^: 0.1 mol L; agitation speed 100 rpm; volume of solution: 10 mL; mass of sorbent = 0.05 g; temperature 298.15 K; I pH 5.5; contact time: 60 min.

## 4 Adsorption Mechanism

The adsorption mechanism consists of a set of physicochemical phenomena that explain how a substance the adsorbate becomes fixed onto the surface of a solid the adsorbent. In the context of removing heavy metal ions through bioadsorbents, this process becomes particularly relevant due to the complexity of the interactions involved and the chemical diversity of these materials.^59^

The main mechanisms associated with the adsorption of heavy metals include: physical adsorption, chemical adsorption, electrostatic interactions, simple diffusion and intraparticle diffusion, hydrogen bonding, redox interactions, complexation, ion exchange, surface precipitation, pore adsorption.^60^

Bioadsorbents such as fruit peels, vegetal residues, algae, and lignocellulosic materials offer both environmental and economic advantages. They also exhibit a high affinity for toxic metals due to the presence of functional groups such as carboxyl, hydroxyl, and amine groups.^61^

Considering the percentage of reduction of 76.8% of the K^+^ ion verified by the XRF table (Table 4), after the contact of the adsorbent with the Pb, it is pointed out the possibility of ion exchange with this cation, the changes in the intensities of the peaks of the carboxylic group of the FTIR, there is the possibility of the formation of complexes of the Pb ions with these groups, as verified by Yuan.^62^

Where there are at least three adsorption mechanisms in the removal of several metals (Pb, Co, Ni and Cu) highlighting the affinity of Pb for the active sites that characterize removal by complexation.^63^ the Ester Groups also has its participation in the complexation.^62^ conformable Figure 12.

**Figure 12:**
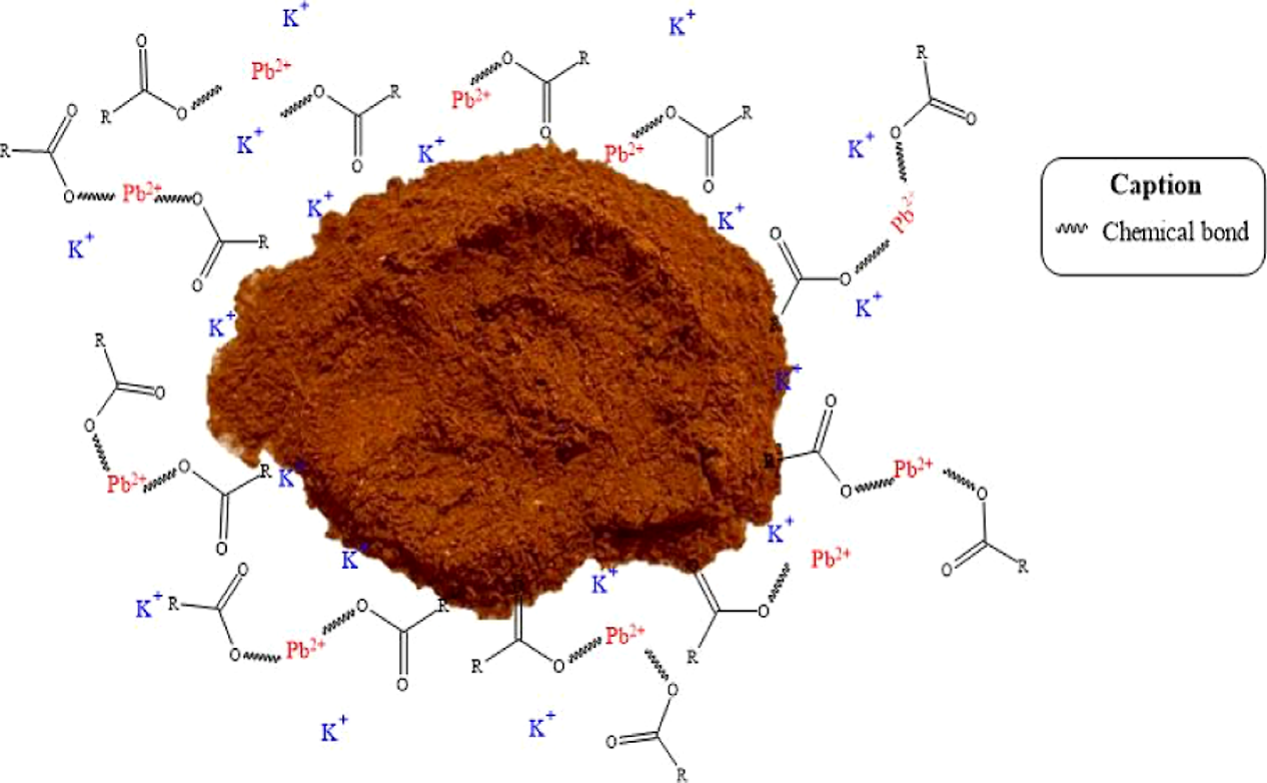
Adsorption of the heavy metal Pb^2+:^ molecular interactions with functional groups.

Effective adsorption via electrostatic interaction is ruled out, as the surface of the adsorbent remains close to zero charge or tends toward a positive surface potential. This condition does not create an attractive environment for Pb^2+^ ions to approach via this mechanism, or at least indicates that electrostatic attraction is not the dominant pathway.

The enthalpy value found is approximately 77.4 kJ mol^–1^, which is near the upper limit associated with weak physisorption bonds, suggesting a predominantly physical nature of the interaction rather than chemisorption.

## 5 Conclusion

Based on the experimental conditions and the results obtained from the adsorption tests, it was concluded that the removal of Pb^2+^ ions using the stem bark of *X. americana* L. was effective in eliminating the pollutant from the aqueous medium. SEM analyzes revealed morphological changes and the formation of probable crystals after treatment, suggesting complexation with the metal. FT-IR spectroscopy indicated alterations in functional groups, including aromatics, carbonyls, and esters. XRF analysis confirmed significant retention of Pb^2+^ in the treated samples (13.12%). Isotherm tests showed the best fit with the Sips model, with a maximum adsorption capacity of 138.2 mg g^–1^ at 298.15 K within 20 min. Notably, even without physicochemical modifications, the material exhibited high *q*_max_ values. The adsorption process was spontaneous and exothermic and fitted well to the pseudo-second order kinetic model. The adsorbent has a point of zero charge (pH_pzc_) of 6.0, and adsorbent dosage did not significantly affect adsorption capacity. Furthermore, the material demonstrated good reusability with minimal loss in efficiency, and the presence of competing ions had no significant influence on adsorption. Thus, the adsorbent proved to be highly suitable for Pb^2+^ removal, exhibiting strong adsorption performance.
